# RUMINATION IN DYADS DURING TIME OF UNCERTAINTY—DAILY LIFE ASSESSMENTS AFTER THE ONSET OF THE COVID-19 PANDEMIC

**DOI:** 10.1093/geroni/igad104.2671

**Published:** 2023-12-21

**Authors:** Elizabeth Zambrano Garza, Rachel Murphy, Maureen Ashe, Kenneth Madden, Anita DeLongis, Denis Gerstorf, Christiane Hoppmann

**Affiliations:** University of British Columbia, Vancouver, British Columbia, Canada; University of British Columbia, Vancouver, British Columbia, Canada; University of British Columbia, Vancouver, British Columbia, Canada; University of British Columbia, Vancouver, British Columbia, Canada; University of British Columbia, Vancouver, British Columbia, Canada; Humboldt University of Berlin, Berlin, Berlin, Germany; University of British Columbia, Vancouver, British Columbia, Canada

## Abstract

Rumination involves repetitive, self-oriented, negative thinking and is known to be detrimental to psychological well-being and health. However, little is known about the extent to which rumination is associated with well-being and health in close relationship partners in older age. Additionally, the pandemic was a time that was characterized by high stress, making it an important context to study rumination. Using daily diary data from 140 Canadian older adults plus a close other of their choice (59% spouses, M = 66.72 years, SD = 13.01 range: 18-87 years, 88% White, 62% women), this project builds on past evidence examining daily life rumination dynamics and extends it to a dyadic perspective beyond couples. For ten days, both dyad members provided evening ratings of daily rumination and affect quality. Multilevel models replicate individual level evidence that higher daily rumination was associated with more daily negative affect and less daily positive affect. Importantly, we also found partner effects such that more close others’ rumination was associated with elevated actors’ negative affect (b = 0.03, p = .038) and reduced actors’ positive affect (b = -0.04, p = .023), suggesting that it is not only one’s own rumination that relates to daily well-being, but also that of a close tie. Findings demonstrate the utility of taking a dyadic perspective on what is typically conceived as an individual-level phenomenon.

